# Surgery *versus* observation in patients 65 years or older with primary hyperparathyroidism: randomized clinical trial

**DOI:** 10.1093/bjsopen/zrag151

**Published:** 2026-10-01

**Authors:** Anders O J Bergenfelz, Mark Thier, Martin Almquist, Jonas P Ranstam, Erik D Nordenström

**Affiliations:** Department of Clinical Sciences, Lund University, Lund, Sweden; Department of Surgery, Skåne University Hospital, Lund, Sweden; Department of Clinical Sciences, Lund University, Lund, Sweden; Department of Surgery, Skåne University Hospital, Lund, Sweden; Department of Orthopaedics, Lund University, Lund, Sweden; Department of Surgery, Skåne University Hospital, Lund, Sweden

**Keywords:** bone density, pHPT, osteoporosis, hypercalcaemia, parathyroidectomy

## Abstract

**Background:**

A substantial portion of surgically treated patients with primary hyperparathyroidism (pHPT) are older than 65 years. The indication for parathyroidectomy in older patients with pHPT and mild hypercalcaemia is unclear. Specifically, it is not known whether parathyroid surgery improves bone mineral density (BMD) in this age group.

**Method:**

Patients 65 years or older with pHPT, ionized calcium not exceeding 1.50 mmol/l, and a BMD Z-score above −2.5 standard deviations who were referred to the Department of Surgery, Lund University Hospital, were included in the study. Follow-up was performed over 2 years. The random allocation sequence to surgery or observation was computer generated. There was no blinding. Primary outcome was BMD of the hip, with the primary endpoint being the change in total hip BMD from baseline to the end of follow-up. Secondary outcome was BMD of the lumbar spine. Continuous variables were analysed using Satterthwaite’s *t*-test. Intention-to-treat analysis of change from baseline was performed using a mixed-model repeated-measures ANOVA (MMRM ANOVA).

**Results:**

A total of 255 patients were screened and 102 were randomized: 50 to observation and 52 to surgery. The median age was 71 (range 65–85) years. For analysis of the primary endpoint, 41 patients in the surgery group and 40 in the observation group were available. Total hip BMD at 2 years increased by a mean of 1.3% (95% confidence interval −0.2% to 2.8%) in the surgery group and decreased by a mean of −1.4% (−2.6% to −0.2%) in the control group (*P* = 0.006). Analysis of total hip BMD at the end of follow-up by MMRM ANOVA showed a difference between the two groups of patients (*P* = 0.006). BMD of the lumbar spine increased by a mean of 4.6% (3.0% to 6.1%) in the surgery group *versus* 0.2% (−1.1% to 1.4%) in the observation group (*P* < 0.001). Analysis of lumbar spine BMD at the end of follow-up by MMRM ANOVA showed a difference between the two groups of patients (*P* < 0.001).

**Conclusion:**

At the 2-year follow-up, patients 65 years or older with pHPT and mild hypercalcaemia demonstrated a slight increase in BMD after parathyroidectomy.

**Registration number:**

NCT01087619 (http://www.clinicaltrials.gov).

## Teaser

In this study of patients with primary hyperparathyroidism (pHPT) older than 65 years and with moderate hypercalcaemia and a Z-score of bone mineral density (BMD) above −2.5 standard deviations, surgery was accompanied by a moderate increase in BMD and, as estimated by FRAX^®^, a modest decreased risk in hip and major osteoporotic fractures over a 10-year period. The results may caution any expansion of the indication for parathyroid surgery or specific medical pHPT treatment in this age group of patients.

## Introduction

Even though contemporary patients with primary hyperparathyroidism (pHPT) are diagnosed and treated with lower calcium values^1,2^, a recent meta-analysis^3^ indicates an increased fracture incidence in these patients. This increased fracture risk is suggested to be reduced by surgery for pHPT^4–7^.

Low bone density, as assessed by dual-energy X-ray absorptiometry (DEXA), is known to be a marker for future fracture risk^8,9^. Over the years, a low bone density, defined as a T-score (bone density compared with the normal range in young healthy adults) below −2.5 standard deviations (s.d.), classified as osteoporosis, has consistently been agreed on as an indication for surgery in pHPT in international guidelines^10–13^.

There is no randomized clinical trial (RCT) that compares surgery for pHPT *versus* clinical observation with fracture incidence as the primary outcome. Instead, bone mineral density (BMD) assessed by DEXA was used in two single-centre RCTs^14,15^ in patients with ‘asymptomatic’ pHPT. Both studies reported some benefit with parathyroidectomy compared with observation. Since these publications more than 20 years ago, only one larger multicentre RCT^16^ has been published, reporting on 191 patients with a mean age of 64 years and a mean value of total calcium of 2.69 mmol/l. For the 116 patients that completed the 2-year follow-up, there was a modest increase in BMD of the femoral neck and lumbar spine in surgically treated patients compared with patients in the observation group^17^.

A substantial proportion of contemporary patients undergoing surgery for pHPT are 65 years or older. According to the 2025 report of the Scandinavian Quality Register for Thyroid, Parathyroid and Adrenal Surgery (SQRTPA)^18^, the mean age of patients undergoing surgery was 64 years.

It is known that morbidity^19^ and frailty^20^ increase with age, with a strong association between them^21^. The impact of parathyroidectomy on bone density specifically in older patients with pHPT is unclear. This RCT was designed to answer this question in patients 65 years or older with moderate hypercalcaemia.

## Methods

This was a single-centre, parallel-group, open-label RCT (acronym *pHPT65*). The study was approved by the Regional Ethical Committee at Lund University; 2010/51 and 2017/211. The study was registered with ClinicalTrials.gov before beginning the study. There was no patient nor public involvement in the planning or conducting of the trial. The trial was conducted in accordance with the principles of the Helsinki Declaration.

Patients referred to the Department of Surgery, Skåne University Hospital-Lund, with hypercalcaemia and raised or, for the calcium level, inappropriately non-suppressed parathyroid hormone (PTH) levels were screened for participation. Eligible patients received written and oral information from a dedicated study nurse. Oral information about the study was repeated to the patient by a surgical specialist during the outpatient visit. All patients included in this study provided written informed consent. The CONSORT checklist is provided as *Supplementary material*.

### Inclusion criteria

Patients 65 years or older referred for first-time surgery of sporadic pHPT.

### Exclusion criteria

- Z-score of BMD less than −2.5 s.d., regardless of site.
- Hypercalcaemia with specific medical treatment (cinacalcet).
- Ionized calcium less than 1.50 mmol/l.
- Patient treated with lithium.
- Inability to understand information or participate in planned follow-up.

### Screening for participation

Based on the written information provided by the referring physician, patients were screened for participation in the study. The screening included BMD, levels of total and ionized calcium, albumin, PTH, creatinine, urinary calcium and 25 (OH) vitamin D_3_. Patients with 25 (OH) vitamin D_3_ levels below 50 nmol/l were prescribed 400IEx2 vitamin D under careful observation of calcium and PTH levels.

Ineligible patients and eligible patients who declined study participation were recorded.

### Randomization

Patients included in the study were randomized 1 : 1 in blocks of six during the outpatient visit to either surgery (intervention) or observation (control). The random allocation sequence was generated using a computer-based random number function in Excel (Microsoft^®^ Excel^®^, Redmond, WA, USA). To implement a concealed treatment allocation, opaque envelopes were used. Neither the patients nor care givers were blinded for the intervention.

### Follow-up

Patients underwent comprehensive follow-up at 1 and 2 years. At the 2-year follow-up, patients in the observation group were offered the choice to undergo parathyroid surgery or further follow-up by the referring physician. Patients who had undergone parathyroidectomy and were normocalcaemic at 2 years were not followed further.

### Intervention group (surgery)

Before surgery, patients were routinely investigated with ultrasound and sestamibi scintigraphy. If the results were equivocal, discordant, or non-informative, patients underwent additional imaging. Surgery was performed under general anaesthesia. Intraoperative PTH (iOPTH) measurements guided the extent of resection of parathyroid tissue. Patients were operated on using a focused or unilateral approach or with bilateral neck exploration, depending on preoperative imaging, results of iOPTH measurement, and clinical findings during surgery. Outcomes such as persistent hyperparathyroidism, medical therapy for hypoparathyroidism, and normocalcaemia at the end of the follow-up were recorded in the SQRTPA; https://sqrtpa.se (later folded into the European Register for Endocrine Surgery, Eurocrine^®^; https://eurocrine.eu).

### Control group (observation)

Patients allocated to the control group were investigated with biochemistry at 3, 6, 9, and 18 months, as well as at the 1- and 2-year follow-up. If ionized calcium increased above 1.50 mmol/l, patients were advised to undergo parathyroid surgery. Furthermore, patients in the control group who demonstrated a Z-score < −2.5 s.d. in BMD of the femoral neck during follow-up were advised to undergo parathyroidectomy.

Hypercalcaemia requiring specific medical treatment also constituted grounds for recommending parathyroidectomy.

After a change into the surgery group (intervention), the patient was followed according to the study protocol.

### Medical treatment before randomization

Patients treated before randomization with bisphosphonates or vitamin D supplements were allowed to continue this medication throughout the study. Newly prescribed medication with bisphosphonates was not allowed during the study period.

### Primary outcome

The primary outcome was the change in BMD of the hip, reported as total hip BMD (g/cm^2^), at 2 years compared with baseline.

### Secondary outcome

Secondary exploratory outcome for this study was the change in BMD (g/cm^2^) of the lumbar spine (L1–L4) at 2 years compared with baseline.

### Other secondary outcomes

Other exploratory secondary outcomes not directly related to the change in bone density will be reported in detail in future manuscripts.

### Bone density

BMD was measured with DEXA. Most investigations were made at Skåne University Hospital in Lund, although some were performed at a hospital closer to the patient’s home. The investigations were performed using GE Lunar Prodigy, GE Lunar iDexa, and Lunar Prodigy Advance (GE Healthcare, Chicago, IL, USA).

BMD is expressed in g/cm^2^, and as s.d. from the mean of a population of young healthy individuals (T-scores) as well as age- and gender-specific s.d. (Z-scores).

The relative change in BMD at 1 and 2 years compared with baseline is reported for the two groups of patients.

BMD measurements were reported for the total hip and lumbar spine (L1–L4), as well as for the femoral neck and the distal third of the radius. For some patients, whole-body BMD was measured. The precision of the measurements has been tested on several occasions in 2010, 2011, 2013, 2021 and 2024. The coefficient of variation ranged from 0.6% to 1.2% for the total hip, from 1.5% to 1.9% for the cervical neck, and from 0.895% to 1.6% for the lumbar spine.

The least significant change (LSC) for total hip BMD has a maximum of 0.031 g/cm^2^. For this study, the LSC for each individual patient was defined as 0.03 g/cm^2^.

### Fracture risk assessment tool (FRAX^®^)

Variables were registered in the study protocol to evaluate the estimated 10-year probability of a hip fracture and any major osteoporotic fracture (a clinical spine fracture, forearm, hip, or shoulder fracture), as calculated according to FRAX^®^ (https://www.fraxplus.org). The following variables were used to calculate the fracture risk at baseline and at the 1-and 2-year follow-up: country of residence (Sweden), age, sex, weight and height, previous fracture, a medical history of a hip fracture in the patient’s biological mother or father, current smoking, glucocorticoid medication, rheumatoid arthritis, secondary osteoporosis, consumption of alcohol at three or more units per day, and BMD of the femoral neck (g/cm^2^, measured by GE-Lunar).

### Clinical and biochemical variables

Age, sex, medical history, including previous fractures, medications, symptoms and signs of pHPT, and variables associated with FRAX^®^ calculation were registered.

Biochemical variables, including total calcium, phosphate, PTH, urinary calcium excretion, osteocalcin, crosslaps, creatinine, and 25 (OH) vitamin D_3_, were analysed on Cobas, Roche (Roche Diagnostics GmbH, Germany) until May 2022, and thereafter on Siemens Atellica (Siemens Healthineers AG, Germany). There was no change in normal ranges, except for PTH; PTH values analysed on Atellica (normal range 1.6–6.9 pmol/l) were converted to PTH Cobas Roche with the equation PTH Roche = PTH Atellica+0.697/1.228.

Serum levels of 1,25 (OH)_2_ vitamin D_3_ were analysed using a radioimmunoassay (INCSTAR Corp., Stillwater, MN, USA) until 2020 and thereafter using the DiaSorin LIAISON^®^ XL 1,25 dihydroxy vitamin D chemiluminescent immunoassay (DiaSorin Inc., Stillwater, MN, USA; marketed by DiaSorin S.p.A., Saluggia, Italy).

Ionized calcium, normal range 1.15–1.33 mmol/l, was analysed using the ABL 800 Flex (Radiometer Medical ApS, Copenhagen, Denmark).

### Power calculation

No previous study has specifically addressed the change in BMD in older patients with pHPT and moderate hypercalcaemia after parathyroid surgery. Thus, the initial power calculation was based on an investigation by Bollerslev *et al.*^16^, which, regarding calcium values, corresponded most closely with the present study. It was preliminary estimated that 80 patients in each arm were needed to show a difference between the intervention and control group at the end of a 2-year period, with a 4% increase in BMD of the femoral neck in the intervention group and a decrease of 4% in the control group, with a power of 80% and alpha of 5%.

However, with slow recruitment and with new information available regarding the precision of DEXA measurement of BMD of the hip, the study group decided to revise the power calculation after first 40 enrolled patients: After the beginning of this study, several publications have shown that BMD of the hip (measured as total hip) has a higher precision compared with BMD of the femoral neck^22,23^, and especially so in overweight and obese patients^24^. These data suggest that total hip BMD measurement may be preferred to BMD of the femoral neck for longitudinal follow-up. Thus, the revised power calculation was performed according to the guidelines for clinical trials^25^ and following the recommendation by Evans^26^. The revised power calculation was performed without any previous data analysis for the primary outcome by the research group. Calculations were based on Friede and Kieser’s^27^ recommendations and used an empirical one-sample s.d. estimate from the 40 patients already randomized, without considering the randomization outcomes.

According to the calculations, the observed s.d. for the 40 patients evaluated by DEXA for total hip BMD was 0.14 g/cm^2^, and the baseline mean BMD was 0.8919 g/cm^2^. To show a 5% increase after 2 years in BMD for the intervention group and a similar decrease in the control group, with 80% power and alpha of 0.05, a sample size of 42 patients in each arm was calculated to be sufficient to evaluate the change in total hip BMD as the primary endpoint. The lumbar spine (L1–L4) was reported as a secondary exploratory outcome. The revised power calculation and the rationale was approved by the Regional Ethical Committee at Lund University 2017/211. The amended study protocol is provided as *Supplementary material*.

### Statistics

Unless otherwise specified, continuous variables are presented in summary form as mean(s.d.). Categorical variables are presented as numbers and percentages. The primary analysis of change from baseline, with adjustment for baseline imbalance, was performed using a mixed-model repeated-measures ANOVA (MMRM ANOVA) with treatment, baseline values, and follow-up as fixed factors and patients as random factor, assuming an independent co-variance structure and using restricted maximum likelihood. Degrees of freedom were calculated according to Satterthwaite’s method. Analyses comparing continuous variables without adjustment were performed using Satterthwaite’s *t*-test. Categorical variables were analysed using χ^2^ tests, or Fisher’s exact test if the lowest expected frequency in the corresponding contingency table was below five. Imputation of missing data was not performed.

Analyses were performed on an intention-to-treat basis, unless otherwise stated. The level of statistical significance was set at 0.05, and statistical analyses were performed using Stata v17.

## Results

The consort flow chart is shown in *Fig. 1*^28^. A total of 255 patients were screened. After applying exclusion criteria and excluding eligible patients who declined to participate, 102 patients were randomized: 50 in the control group (observation) and 52 in the intervention group (surgery). No patients had known hereditary pHPT. The median age of patients in the cohort was 71 (range 65–85) years. The mean(s.d.) value of ionized calcium was 1.40(0.05) mmol/l (mean total calcium 2.62 mmol/l). Although all patients had hypercalcaemia at the time of referral, eight patients (7.8%) had a high normal ionized calcium level as measured at baseline after randomization, ranging from 1.28 mmol/l to 1.33 mmol/l. The mean(s.d.) level of PTH was 10.1(2.9) pmol/l at baseline, with 11 patients (10.8%) with levels within the normal range. Some 78 patients (76.5%) were women, and 36 patients (35.3%) had sustained at least one fracture before the study. The mean(s.d.) Z-score for total hip was −0.1(0.9), and no patients had, by definition, a Z-score below −2.5 s.d. The mean(s.d.) T-score for total hip was −1.3(1.0) and only five patients had a T-score of total hip below −2.5 s.d..

**Figure 1:**
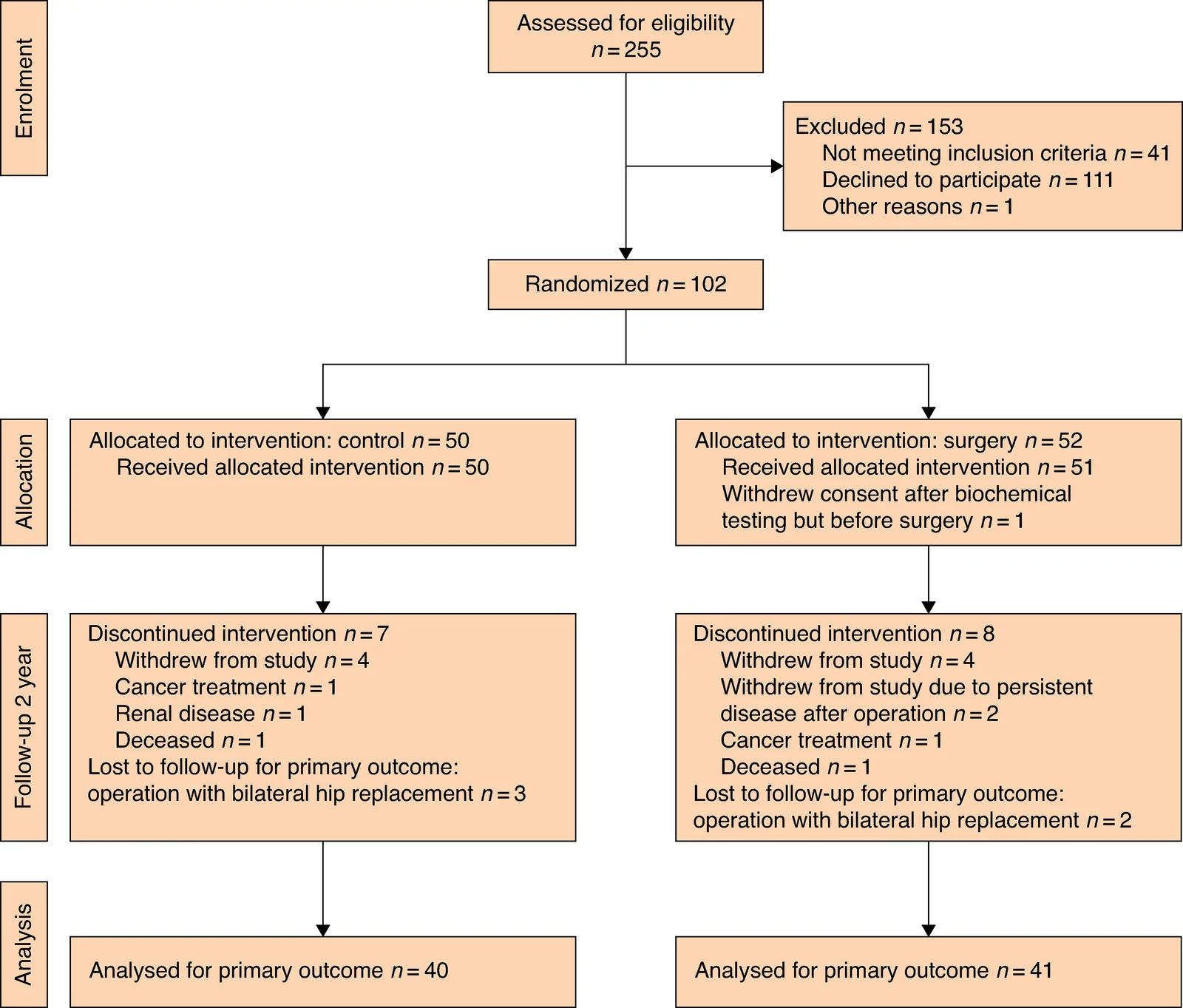
CONSORT diagram of the patients screened, randomized, and followed during the pHPT65 study A total of 86 patients completed the study, and 81 of these patients were available for analysis of the primary outcome and 86 patients for the secondary outcome.

The patients were randomized between 29 September 2010 and 28 September 2023, with a 26-month break (January 2020 until March 2022) during the COVID-19 pandemic, due to hospital regulations. Clinical variables in the two randomized groups are shown in *Table 1*. Biochemical data at baseline and at the 1- and 2-year follow-up for the two groups of patients and outcomes at the end of follow-up as analysed with MMRM ANOVA, are shown in *Table 2*. At the end of the study period, ionized calcium, total calcium, phosphate, PTH, urinary calcium, crosslaps, osteocalcin, creatinine, 25 (0H) vitamin D_3_, and 1,25 (OH)_2_ vitamin D_3_ differed between the two groups of patients.

**Table 1:** Clinical variables at baseline of patients randomized to observation (control group) or surgical treatment (intervention group) in patients 65 years or older with primary hyperparathyroidism

	Observation group (*n* = 50)	Surgery group (*n* = 52)
**Sex**		
Women	36 (72%)	42 (81%)
Men	14 (28%)	10 (19%)
Age, years, mean(s.d.)	72.1(4.4)	72.1(4.6)
BMI, mean(s.d.)	28.7(5.2)	28.7(4.3)
Vitamin D supplements	12 (24%)	11 (21%)
Bisphosphonate	2 (4%)	6 (12%)
Oral steroid treatment	0 (0%)	1 (2%)
Diabetes mellitus	8 (16%)	11 (21%)
Previous fracture	18 (36%)	18 (35%)

Values are *n* (%), unless otherwise specified. s.d., standard deviation; BMI, body mass index.

**Table 2:** Biochemical variables at baseline and at the 1- and 2-year follow-up for patients randomized to observation or surgical treatment in patients 65 years or older with primary hyperparathyroidism

	Baseline		1 year		2 years		P*
	Observation	Surgery	Observation	Surgery	Observation	Surgery	
Ionized calcium (mmol/l)	1.40(0.05)	1.40(0.05)	1.38(0.05)	1.23(0.04)	1.39(0.06)	1.23(0.05)	< 0.001
	(*n* = 50)	(*n* = 52)	(*n* = 46)	(*n* = 44)	(*n* = 43)	(*n* = 43)	
Total calcium (mmol/l)	2.61(0.10)	2.62(0.09)	2.59(0.10)	2.32(0.10)	2.61(0.11)	2.34(0.11)	< 0.001
	(*n* = 50)	(*n* = 52)	(*n* = 45)	(*n* = 43)	(*n* = 43)	(*n* = 42)	
PTH (pmol/l)†	10.2(2.7)	10.0(3.2)	10.0(3.5)	5.7(2.3)	10.1(3.3)	5.4(2.8	< 0.001
	(*n* = 50)	(*n* = 52)	(*n* = 46)	(*n* = 44)	(*n* = 43)	(*n* = 43)	
Phosphate (mmol/l)	0.81(0.14)	0.85(0.13)	0.82(0.12)	0.99(0.15)	0.81(0.12)	1.03(0.14)	< 0.001
	(*n* = 50)	(*n* = 52)	(*n* = 46)	(*n* = 44)	(*n* = 42)	(*n* = 43)	
Creatinine (umol/l)	76(19)	78(20)	78(18)	81(22)	77(19)	82(22)	0.044
	(*n* = 50)	(*n* = 52)	(*n* = 46)	(*n* = 44)	(*n* = 43)	(*n* = 43)	
Osteocalcin (ug/l)	31(20)	37(20)	27(13)	21(13)	28(13)	23(17)	< 0.001
	(*n* = 49)	(*n* = 51)	(*n* = 46)	(*n* = 44)	(*n* = 42)	(*n* = 40)	
Crosslaps (ng/l)	487(236)	576(258)	492(306)	320(202)	529(280)	378(249)	< 0.001
	(*n* = 48)	(*n* = 52)	(*n* = 45)	(*n* = 43)	(*n* = 42)	(*n* = 40)	
25-OH vitamin D_3_ (nmol/l)	58(21)	58(21)	63(21)	67(24)	57(20)	74(41)	< 0.001
	(*n* = 52)	(*n* = 50)	(*n* = 40)	(*n* = 43)	(*n* = 42)	(*n* = 40)	
1.25 (OH)_2_ vitamin D_3_ (pmol/l)	95(42)	100(37)	110(60)	72(30)	98(43)	81(35)	0.034
	(*n* = 50)	(*n* = 44)	(*n* = 35)	(*n* = 35)	(*n* = 36)	(*n* = 38)	
Urinary calcium mmol per l per 24 hours	4.52(3.11)	4.36(2.79)	5.24(4.39)	2.62(2.14)	5.20(3.67)	2.92(2.15)	0.001
	(*n* = 47)	(*n* = 50)	(*n* = 44)	(*n* = 38)	(*n* = 39)	(*n* = 35)	

Values are mean(standard deviation). Normal ranges: total calcium 2.15–2.50 mmol/l; phosphate, men 0.7–1.4 mmol/l, women 0.8–1.5 mmol/l; creatinine, men 60–105 umol/l, women 45–90 µmol/l; osteocalcin 10–43 µg/l; crosslaps, age 51–70 < 710 ng/l, age > 70 < 730 ng/l; 25 (OH) vitamin D_3_ recommended level > 75 mmol/l; 1,25 (OH)_2_ vitamin D_3_ 48–190 pmol/l; urinary calcium/24 hours 2.5–7.5 mmol; ionized calcium 1.15–1.33 mmol/l, PTH 1.6–6.9 pmol/l. †PTH was analysed on Cobas instrument-Roche until May 2022, and thereafter on Atellica instrument-Siemens with normal range 2.0–8.5 pmol/l. PTH values analysed on Atellica was converted to PTH analysed on Cobas Roche with the equation: PTH Roche = PTH Atellica+0.697/1.228. *MMRM ANOVA.

During the first year of follow-up, 10 patients discontinued to study; 2 patients due to cancer treatment, and 2 patients (4%) with persistent disease after parathyroid surgery declined further participation in the study. Of note, two patients died, three patients declined to continue the study for personal reasons, and one patient discontinued the study due to renal disease. Furthermore, at the 1-year follow-up, two patients declined scheduled follow-up investigations due to the COVID-19 pandemic, although one of these patients completed the BMD measurements. Both these patients completed the follow-up at 2 years. Thus, the 1-year follow up was performed in 91 patients, although one of these patients only completed the bone density investigation.

After the 1-year follow-up, a further six patients discontinued the study. In total, 86 patients completed the 2-year follow-up. Clinical and biochemical data and BMD on the 86 patients that completed the study, and the 16 patients that discontinued the study before the 2-year follow-up, are shown in *Table S1*.

As 5 of the 86 patients that completed the 2-year follow-up had undergone a bilateral hip replacement, the primary endpoint of the study could be evaluated in 81 patients (40 in the control group and 41 in the intervention group).

In the observation group, two patients underwent parathyroidectomy during the study due to an increase in ionized calcium > 1.50 mmol/l. Both patients were normocalcaemic after surgery and continued the study according to the study protocol. These patients are included in the surgery group in the ‘*as treated*’ analysis for the primary outcome.

Parathyroid surgery (including the two patients in the control group) was performed as bilateral neck exploration (21), focussed operation (26), and unilateral neck exploration (5). For two operations, the type of parathyroid surgery was not registered. Eight patients underwent concomitant thyroid operation (hemithyroidectomy in four and total thyroidectomy in four). The mean(s.d.) excised parathyroid gland weight was 0.62(0.42) grams. Some 38 patients were diagnosed with parathyroid adenoma and 12 patients with multiglandular parathyroid neoplasia. In four patients the histological diagnosis was unclear. At the 2-year follow-up, all patients had normal levels of ionized calcium, except for one patient with asymptomatic hypocalcaemia and normal PTH (1.07 mmol/l and 4.3 pmol/l, respectively). One patient had an ionized calcium level at the upper normal range, but with PTH in the lower range (1.30 mmol/l and PTH 2.3 pmol/l, respectively). Two patients (4%) were treated after surgery with active vitamin D due to hypoparathyroidism (one of these patients was operated due to multiglandular disease and one patient was subjected to concomitant thyroidectomy due to thyrotoxicosis).

### Bone density results

The results of BMD measurements (g/cm^2^), T-scores, and Z-scores at baseline and at the 1- and 2-year follow-up are shown in *Table 3*.

**Table 3:** Absolute bone mineral density (g/cm^2^), T-scores, and Z-scores at baseline and at the 1- and 2-year follow-up in patients with primary hyperparathyroidism randomized to observation or surgery

	Baseline		1 year		2 years	
	Observation	Surgery	Observation	Surgery	Observation	Surgery
Total hip (g/cm^2^)	0.884(0.130)	0.844(0.132)	0.881(0.130)	0.853(0.145)	0.880(0.129)	0.854(0.152)
	(*n* = 49)	(*n* = 49)	(*n* = 44)	(*n* = 43)	(*n* = 40)	(*n* = 41)
Total hip T-score	−1.1(0.9)	−1.4(1.0)	−1.2(1.0)	−1.4(1.0)	−1.2(1.0)	−1.3(1.1)
	(*n* = 49)	(*n* = 49)	(*n* = 44)	(*n* = 43)	(*n* = 40)	(*n* = 41)
Total hip Z-score	−0.0(0.9)	−0.2(0.9)	−0.0(0.9)	−0.1(1.0)	0.0(0.9)	0.0(1.0)
	(*n* = 49)	(*n* = 49)	(*n* = 44)	(*n* = 43)	(*n* = 40)	(*n* = 41)
Cervical neck (g/cm^2^)	0.816(0.119)	0.792(0.119)	0.803(0.110)	0.801(0.132)	0.803(0.108)	0.796(0.138)
	(*n* = 49)	(*n* = 49)	(*n* = 44)	(*n* = 43)	(*n* = 40)	(*n* = 41)
Cervical neck T-score	−1.7(0.9)	−1.8(0.8)	−1.8(0.8)	1.8(0.9)	−1.8(0.8)	−1.8(1.0)
	(*n* = 49)	(*n* = 49)	(*n* = 44)	(*n* = 43)	(*n* = 40)	(*n* = 41)
Cervical neck Z-score	−0.3(0.8)	−0.4(0 .8)	−0.3(0.7)	−0.2(0.9)	−0.3(0.8)	−0.2(0.9)
	(*n* = 49)	(*n* = 49)	(*n* = 44)	(*n* = 43)	(*n* = 40)	(*n* = 41)
Lumbar spine (g/cm^2^)	1.127(0.209)	1.057(0.192)	1.121(0.199)	1.099(0.198)	1.123(0.196)	1.115(0.207)
	(*n* = 50)	(*n* = 52)	(*n* = 46)	(*n* = 45)	(*n* = 43)	(*n* = 43)
Lumbar spine T-score	−0.6(1.7)	−1.1(1.5)	−0.6(1.6)	−0.8(1.6)	−0.6(1.5)	0.7(1.6)
	(*n* = 50)	(*n* = 52)	(*n* = 46)	(*n* = 45)	(*n* = 43)	(*n* = 43)
Lumbar spine Z-score	0.5(1.5)	0.0(1.3)	0.4(1.4)	0.4(1.4)	0.4(1.4)	0.6(1.4)
	(*n* = 50)	(*n* = 52)	(*n* = 46)	(*n* = 45)	(*n* = 43)	(*n* = 43)
Distal radius (g/cm^2^)	0.775(0.131)	0.732(0.133)	0.765(0.139)	0.724(0.146)	0.746(0.139)	0.705(0.153)
	(*n* = 46)	(*n* = 51)	(*n* = 46)	(*n* = 44)	(*n* = 43)	(*n* = 43)
Distal radius T-score	−1.6(1.1)	−1.9(1.3)	−1.7(1.2)	−2.0(1.3)	−1.8(1.2)	−2.2(1.4)
	(*n* = 46)	(*n* = 51)	(*n* = 46)	(*n* = 44)	(*n* = 43)	(*n* = 43)
Distal radius Z-score	0.0(1.0)	−0.2(1.1)	0.1(1.1)	−0.2(1.1)	−0.1(1.1)	−0.2(1.2)
	(*n* = 46)	(*n* = 51)	(*n* = 46)	(*n* = 44)	(*n* = 43)	(*n* = 43)
Total body (g/cm^2^)	1.072(0.094)	1.053(0.107)	1.090(0.107)	1.051(0.122)	1.082(0.113)	1.037(0.133)
	(*n* = 32)	(*n* = 31)	(*n* = 45)	(*n* = 34)	(*n* = 41)	(*n* = 39)
Total body T-score	−0.8(1.1)	−1.0 (1.2)	−0.5 (1.0)	−0.9 (1.1)	−0.6 (1.0)	−0.8 (1.2)
	(*n* = 32)	(*n* = 30)	(*n* = 45)	(*n* = 34)	(*n* = 41)	(*n* = 39)
Total body Z-score	−0.1(1.0)	−0.3(1.0)	0.23(1.03)	−0.0(0.9)	0.2(1.0)	0.2(1.0)
	(*n* = 32)	(*n* = 31)	(*n* = 45)	(*n* = 34)	(*n* = 41)	(*n* = 39)

Values are mean(standard deviation).

### Primary outcome

A total of 81 patients were available for the *intention-to-treat* analysis of the primary endpoint at the 2-year follow-up (41 in the surgery group and 40 in the observation group).

BMD reported as total hip had increased by mean 1.3% (95% confidence interval (c.i.) −0.2% to 2.8%) in the surgery group and decreased by mean −1.4% (−2.6% to −0.2%) in the control group, with a mean difference between the two groups of −2.7% (−4.6% to −0.8%) (*P* = 0.006). In the *as-treated analysis*, which included 43 patients in the surgery group and 38 patients in the control group, the mean increase in BMD was 1.3% (−0.2% to 2.7%) in the surgery group and, in the observation group, BMD decreased by mean −1.5% (−2.8% to −0.3%), with a mean difference of −2.8% (−4.7% to −0.9%) between the two groups of patients (*P* = 0.004).

The change in BMD (g/cm^2^) at the hip was analysed by the MMRM ANOVA, and the result is shown in *Fig. 2a*. At the end of the follow-up, BMD for total hip differed between the two groups of patients, with the surgical group having higher BMD than the observation group.

**Figure 2:**
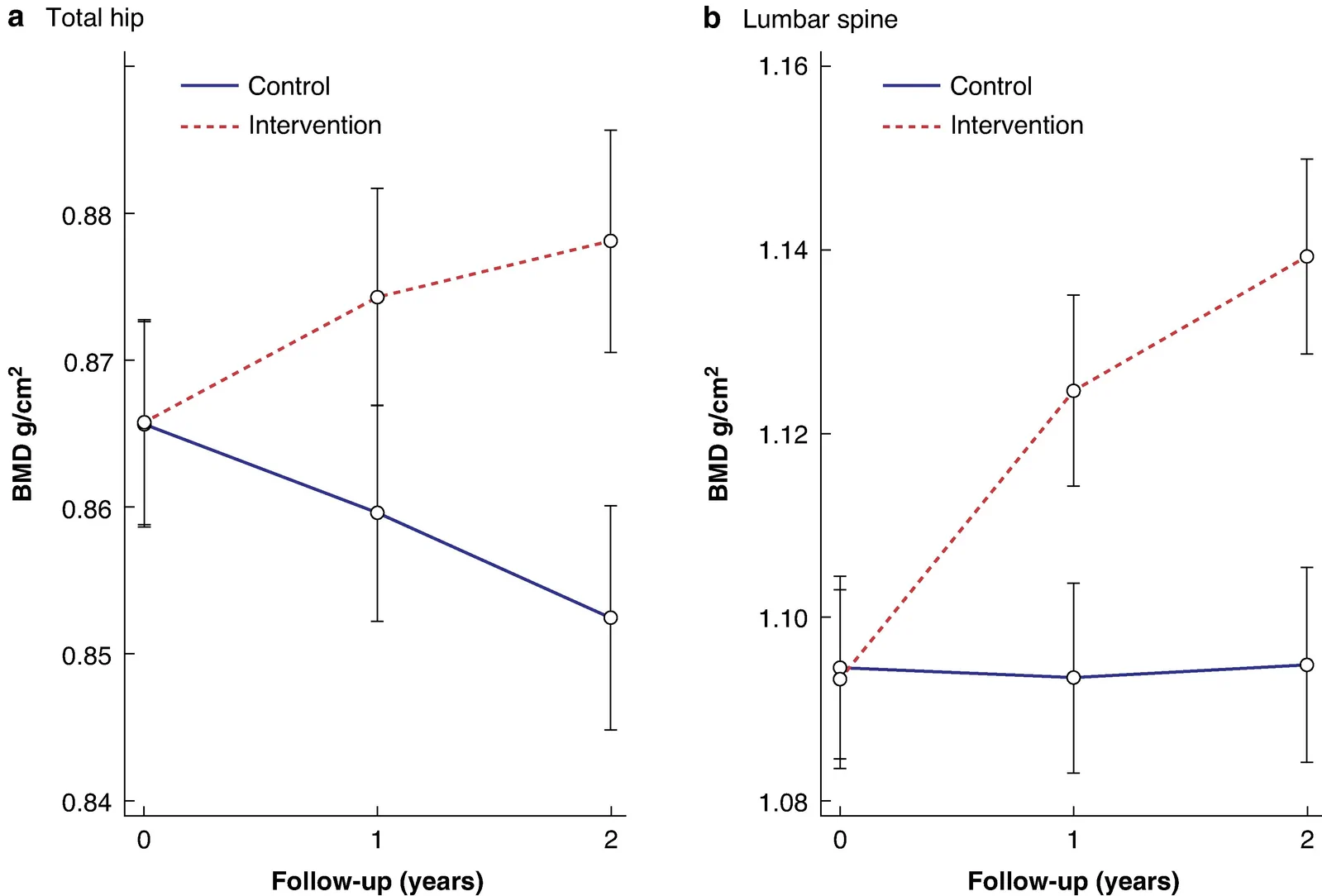
BMD (g/cm^2^) during study period analysed by mixed-model repeated-measures ANOVA for the two patient groups **a** total hip (*P* < 0.001), **b** lumbar spine (*P* < 0.001). Mean (95% confidence intervals) are shown. *P* values at the end of the study period. BMD, bone mineral density.

For individual patients, an increase in BMD, corresponding to an LSC of ≥ 0.03 g/cm^2^, was seen in 4 of 40 patients in the control group and 16 of 41 patients in the intervention group (*P* = 0.002).

### Secondary exploratory outcome

Secondary outcome was evaluated by measurement of BMD (g/cm^2^) at the lumbar spine (L1–L4) for both groups of patients.

The analysis included 86 patients (43 in the surgery group and 43 in the control group). BMD at the 2-year follow-up increased by a mean of 4.6% (95% c.i. 3.0% to 6.1%) in the surgery group and by 0.2% (−1.1% to 1.4%) in the observation group, with a mean difference between the two groups of −4.4% (−6.4% to −2.4%) (*P* < 0.001). The MMRM ANOVA analysis showed that BMD was higher for the intervention group than for the control group (*Fig. 2b*).

### Women and men

The increase in BMD for the total hip and lumbar spine for the intervention group compared with the control group was similar for women and men at the end of the study period, albeit with more uncertainty for men due to small numbers (*Table S2*).

### Other bone density analysis and measurements

The MMRM ANOVA analysis for Z-scores and T-scores for total hip and the lumbar spine are shown in *Figs 3a,b* and *4a,b*, respectively. The intervention group had higher Z-scores and T-scores for total hip and the lumbar spine.

**Figure 3:**
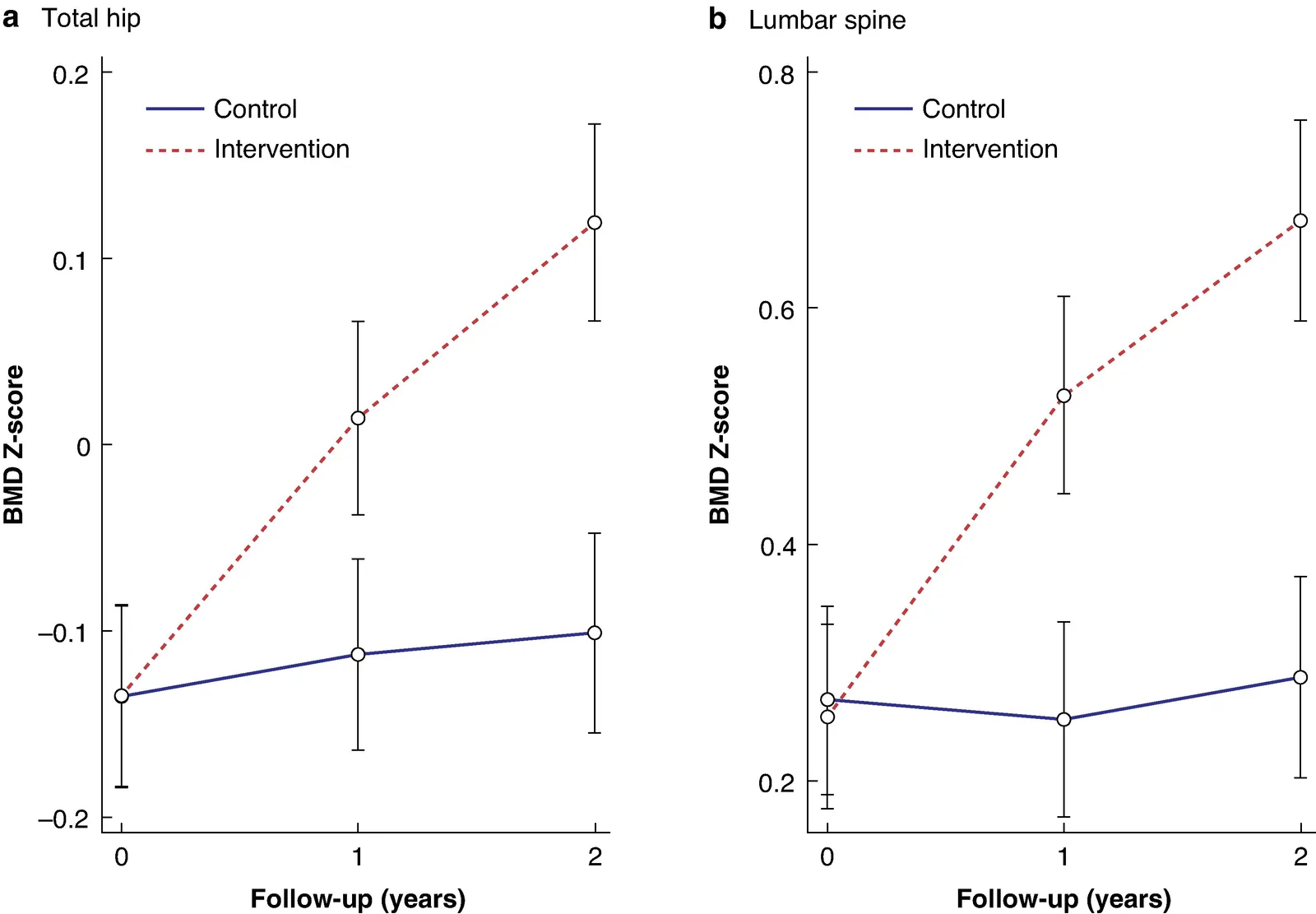
Bone density expressed as Z-scores during study period analysed by mixed-model repeated-measures ANOVA for the two patient groups **a** total hip (*P* < 0.001), **b** lumbar spine (*P* < 0.001). Mean (95% confidence intervals) are shown. *P* values at the end of the study period. BMD, bone mineral density.

**Figure 4:**
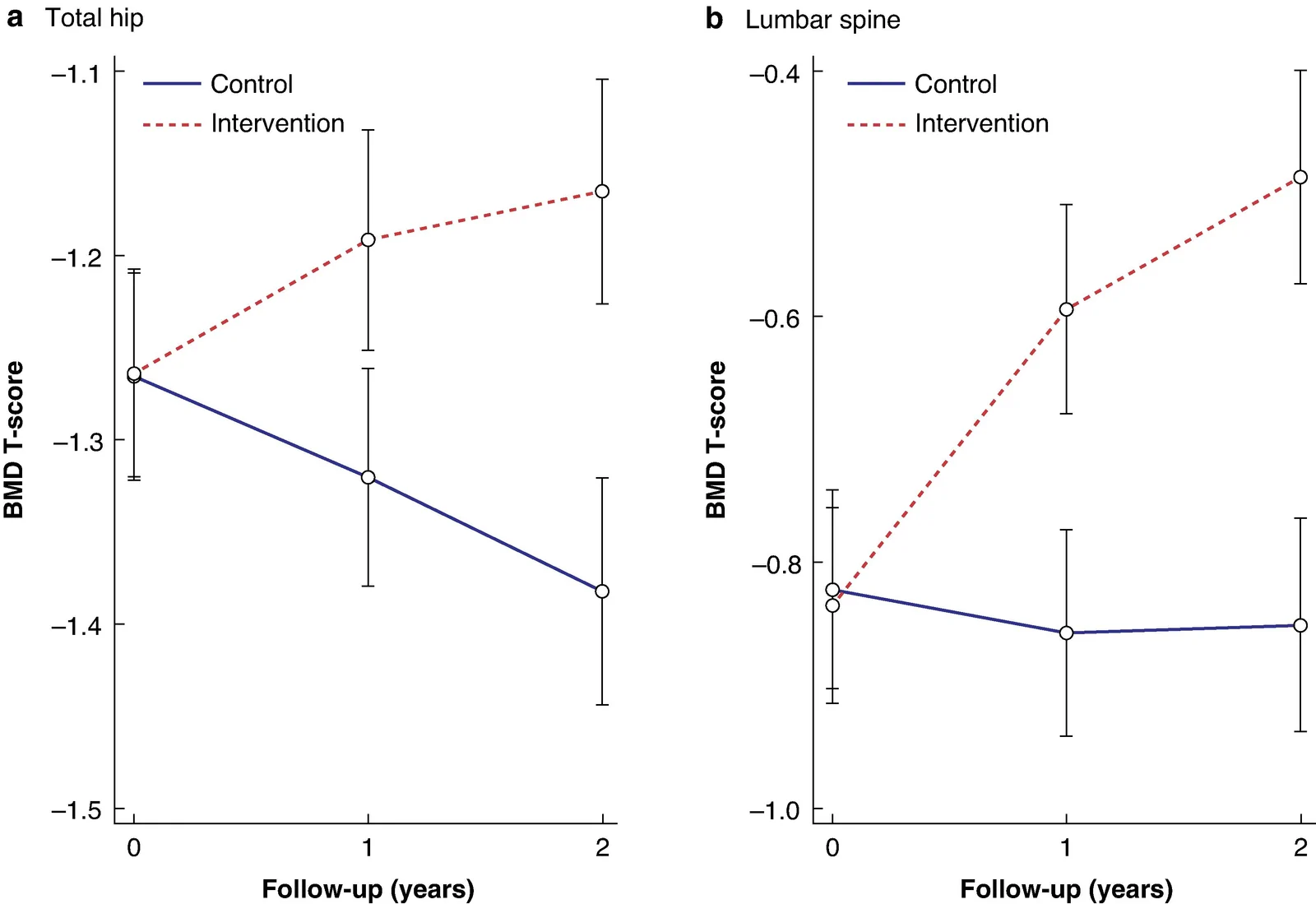
Bone density expressed as T-scores during study period analysed by mixed-model repeated-measures ANOVA for the two patient groups **a** total hip (*P* < 0.001), **b** lumbar spine (*P* < 0.001). Mean (95% confidence intervals) are shown. *P* values at the end of the study period. BMD, bone mineral density.

BMD measured in the femoral neck increased at 2 years by mean 1.2% (95% c.i. −0.7% to 3.0%) in the surgery group and decreased in the control group by −1.1% (−2.7% to 0.5%), with a mean difference between the two groups of −2.2% (−4.6% to 0.1%) (*P* = 0.064). At the distal radius, the analysis of 82 patients (40 the control group and 42 in the intervention group), showed a decrease in BMD in the control group by mean −4.0% (−5.2% to −2.9%) and by mean −3.5% (−5.3% to −1.6%) in the surgery group, with a mean difference between the groups of −0.5% (−2.7% to 1.6%) (*P* = 0.618).

The MMRM ANOVA analysis of bone density of the femoral neck showed higher BMD, Z-scores, and T-scores for the surgical group compared with the control group (*P* < 0.001 for all variables; *Fig. S1a–c*). The MMRM ANOVA analysis of bone density of the distal radius showed no statistically significant difference for BMD (*P* = 0.084)) and T-scores (*P* = 0.844) between the two patient groups, although there was a statistically significant difference in Z-scores (*P* = 0.006); *Fig. S2a–c*.

Total body BMD was measured in only 51 patients. Results are shown in *Table 3*.

### Fracture risk assessment assessed by FRAX^®^

The change in estimated fracture risk at the end of follow-up as analysed by MMRM ANOVA for hip and any major osteoporotic fracture risk are shown in *Fig. 5a*,*b*. At the end of follow-up, the estimated risk for sustaining a hip fracture or any osteoporotic fracture was lower in patients undergoing parathyroidectomy compared with patients in the observation group (*P* = 0.009 and *P* = 0.005, respectively).

**Figure 5:**
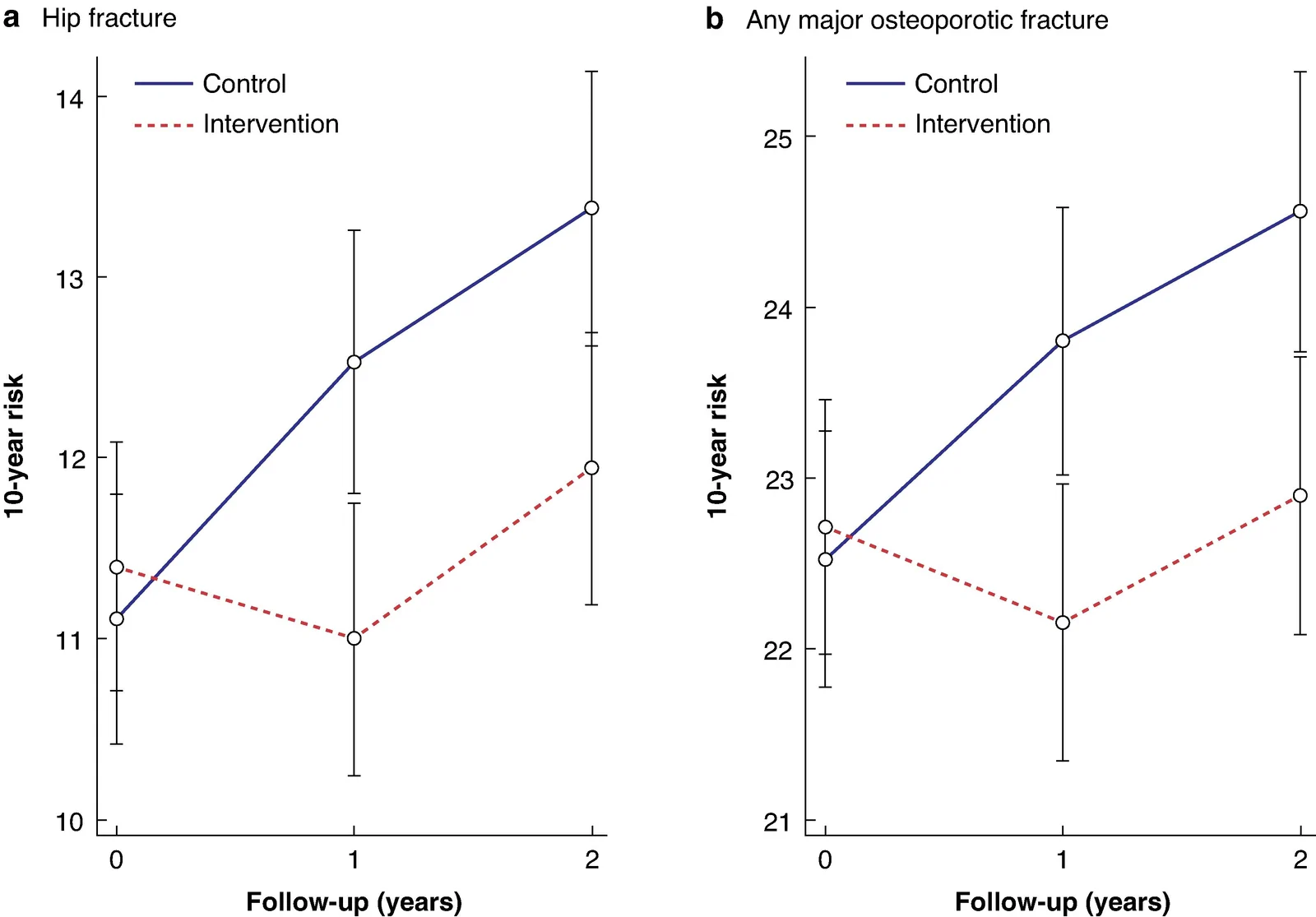
Mixed-model repeated-measures ANOVA used to analyse 10-year risk (%) of sustaining fractures. **a** hip fracture (*P* = 0.009), **b** any major osteoporotic fracture (*P* = 0.005) according to the FRAX® instrument. Mean (95% confidence intervals) are shown. *P* values at the end of the study period.

At the 2-year follow-up, the mean risk for hip fracture within 10 years was 13.4% (95% c.i. 12.6% to 14.1%) for the control group and 11.9% (11.2% to 12.7%) for the intervention group. The mean risk for any major osteoporotic fracture within 10 years was 24.6% (23.7% to 25.4%) for the control group and 22.9% (22.1% to 23.7%) for the intervention group.

### Subgroup analysis

Not all patients underwent follow-up DEXA measurements using the same GE Lunar device. Therefore, a subgroup analysis was performed including only patients whose DEXA measurements throughout the study were obtained using the same scanner. This analysis excluded 41 observations. The MMRM analysis showed no significant differences between the results for the entire cohort and those for the subgroup of patients examined using the same GE Lunar scanner, except that the smaller sample size resulted in greater uncertainty in the estimates (*Figs S3–S6*).

### End of follow-up

At the end of the study, patients in the control group had the option of further follow-up by the referring physician or proceed with parathyroid surgery. Only one patient chose surgery. However, to date on 20 November 2025, a further three patients in the control group have been referred for surgery. One of these opted for follow-up only, and two patients have undergone surgery. Thus, in total, of the 50 patients randomized to observation in this study, 5 (10%) patients underwent surgery during or after the 2-year study period.

## Discussion

The main finding of the present investigation was that BMD, measured as total hip, increased modestly in the surgery group compared with the control group. The mean difference between the two groups was 2.7% at the 2-year follow-up. Total hip BMD was used as the primary endpoint due to its higher precision compared with femoral neck^22,23^. It has also been suggested that measurement of total hip may outperform femoral neck for prediction of major osteoporotic fractures, possibly because it evaluates more bone, especially trabecular bone^29^.

In the lumbar spine, BMD in patients undergoing surgery increased by 4.6%, whereas BMD in the observation group did not differ statistically from baseline.

The differences between the two groups for the primary and secondary outcomes were confirmed by the MMRM ANOVA analysis.

However, absolute BMD of the distal radius at the end of follow-up showed a decrease in both groups of patients, with no major difference between them.

Taken together, these results suggest that there is a modest influence on BMD of the hip and lumbar spine after surgery for pHPT in the age group studied, in agreement with a recent meta-analysis^30^. The impact of parathyroidectomy seems to be more pronounced in areas with cancellous bone.

In summary, these results may caution any expansion of the indication for parathyroid surgery or specific medical pHPT treatment in this age group of patients.

However, longer-term follow-up may provide additional information regarding the need for subsequent surgery for pHPT in the control group, as well as fracture incidence and deaths in both groups. Given the slow recruitment in this study, future multicentre RCTs in this field should be encouraged.

In the Scandinavian SIPH study, an RCT of patients with asymptomatic pHPT, with a mean age of 63 years and baseline calcium levels of 2.68 mmol/l and 2.70 mmol/l in the observation group and surgery group, respectively, BMD at the 2-year follow-up in the surgical group increased by 2.7% in the lumbar spine and by 1.1% in the femoral neck. The difference between the two groups was 4.84% for the lumbar spine and 2.02% for the femoral neck at 2 years, compared with baseline values^17^. Compared with the present study, the results are broadly similar, the main difference being that patients in the present cohort are approximately 10 years older. Interestingly, in a long-term follow-up of the patients in the SIPH study performed after 10 years, a significant effect of parathyroidectomy was found in bone density for all compartments compared with observation, including the distal radius^31^. This is of interest as the effect on bone density of parathyroidectomy at the distal radius has been difficult to establish with short follow-up^17^.

Patients with pHPT have been shown to have a higher incidence for any fracture at the time of surgery^3,6^ or at diagnosis^32^. Specifically, the fracture incidence is increased in the forearm^3,6^, vertebrae^3^, and hip^32^. In conservatively managed patients with pHPT, the fracture incidence is increased after diagnosis compared with controls, regardless of calcium and PTH levels^32^. For any given BMD, the risk for hip and other major osteoporotic fractures increased in patients with pHPT compared with controls^32^.

The impact of parathyroid surgery on fracture risk in patients 65 years or older with pHPT, compared with conservatively treated patients, was specifically evaluated in a retrospective observational study by Medicare insurance claims in the US^33^. The two patient groups differed, and to reduce selection bias of treatment propensity score weighting was used for the statistical analysis. In the multivariable analysis, that study found that parathyroidectomy was associated with a lower adjusted rate of any fracture (hazard ratio 0.78) and hip fracture (hazard ratio 0.76), and this was sustained for up to 10 years. The weakness of that study was that disease severity, as assessed by calcium and PTH levels, was not available. Similarly, results of BMD measurements were lacking. This may lead to residual confounders not accounted for, which may influence the association between parathyroidectomy and the risk reduction in fracture incidence.

In the present study, the risk of hip fracture and any major osteoporotic fracture was estimated by the FRAX^®^ assessment tool. The FRAX^®^ estimated risk for sustaining a hip fracture, or any major osteoporotic fracture, was lower in the group of patients who underwent parathyroidectomy compared with the control patients, although the difference, as shown in *Fig. 5a*,*b*, was modest.

An important point is whether the present cohort reflects appropriately surgically treated patients in Sweden and Europe. Based on data of 9548 registered patients by 99 clinics in 14 countries in Eurocrine^®^, a major database for endocrine surgical procedures in Europe, the median age of patients with pHPT in Sweden was 64 years and the median preoperative calcium value was 2.74. For the total European cohort of patients, the median age was 62 years, and the median preoperative calcium value was 2.75 mmol/l^34^. Thus, the patients in the present study were approximately a decade older compared with the European cohort, and the mean total calcium value was lower; none of the patients included in the present investigation had a preoperative ionized calcium value above 1.50 mmol/l (approximately total calcium 2.90 mmol/l). Furthermore, although the excised gland weight in the present study was lower compared with the European study, the cure rate was similar.

There are some weaknesses in the present study. The trial took a long time to complete. This was partly due to the COVID-19 pandemic, with a more than 2-year break in recruitment. Even more importantly, of the 255 patients screened for participation, 111 eligible patients declined to participate. This number should be put in the context of the 102 patients that were randomized. The reason for declining to participate were several, but most prominent was that many patients had already decided to undergo surgery after discussion by the referring physician. It is also noteworthy that 16 of 102 patients did not complete the 2-year follow-up, reflecting the studied age group. However, there is no evidence that this skewed the results (*Table S1*).

Although most bone density investigations were performed at Skåne University Hospital in Lund, some were done at other hospitals, and three different GE-DEXA machines were used during the study period. These have been tested on several occasions with no major differences among them and with good accuracy, and cross calibration was not deemed necessary. The study results were upheld by a subgroup analysis of patients whose DEXA measurements were obtained using the same GE DEXA equipment. Furthermore, results are also presented with T-scores and Z-scores, and the results between the two study groups remained consistent.

The risk for hip fracture and any major osteoporotic event was estimated by the FRAX^®^ instrument. It may be argued that this instrument does not capture the change in bone microarchitecture in patients with pHPT. In agreement, it has been suggested that the fracture risk is increased in patients with pHPT independently of BMD^32^. It is not known, however, how much of the residual increased fracture risk in pHPT could be attributed to a change in bone architecture. A recent 10-year follow-up of a Danish RCT comparing parathyroidectomy with observation in patients with mild pHPT did not show a difference in the risk for fractures between the two groups of patients, although the numbers were small^31^. This agrees with the modest difference in fracture risk estimated by FRAX^®^ in the present investigation.

The strengths of the study are that consecutive patients (except for the time for the COVID-19 pandemic) were included in the study. Information and follow-up of patients in the study was performed by a dedicated study nurse and endocrine surgical specialists. A further strength in the design of the study is that patients in the control group had the choice after completion of the study to be followed by the referring doctor or undergo pHPT surgery. Only one patient opted for surgery, although a further two patients have since had surgery.

## Supplementary Material

zrag151_Supplementary_Data

## Data Availability

The data, methods used in the analysis, and materials used to conduct the research will be made available by request to the corresponding author to any researcher for the sole purpose of reproducing the results.
