# Supplementary material for: Surgery versus observation in patients 65 years or older with primary hyperparathyroidism: randomized clinical trial
Source: BJS Open. 2026 Oct 1;10(5):zrag151. doi: 10.1093/bjsopen/zrag151 (PMC13627735; doi:10.1093/bjsopen/zrag151)
Supplement: Supplementary file 1 [file zrag151_supplementary_data.docx]

**Title**

**A parallel randomised controlled trial of surgery versus observation in patients 65 years or older with primary hyperparathyroidism**

**Authors:**

Anders OJ Bergenfelz^1^, Mark Thier^2^, Martin Almquist ^1,2^, Jonas P Ranstam^4^, Erik D Nordenström^2^.

^1^Department of Clinical Sciences, Lund University, Lund, Sweden

^2^Department of Surgery, Skåne University Hospital Lund, Sweden

^3^Department of Endocrinology, Skåne University Hospital Lund, Sweden

^4^ Department of Orthopaedics, Lund University, Lund, Sweden

**Corresponding author.**

Anders Bergenfelz

anders.bergenfelz@med.lu.se

Grisslevägen 4

277 32 Lund

Sweden

ORCID ID: 0000-0002-8355-6025

**Supplementary Materials – Index**

| **Supplementary Appendixes** |  |
| --- | --- |
| Consort Checklist | *pag.2-4* |
| Study protocol | *pag.5-11* |
| **Supplementary Figures and Tables** |  |
| Suppl. Table 1 | *pag.12* |
| Suppl. Table 2 | *pag.13* |
| Suppl. Fig 1a-c | *pag.14* |
| Suppl. Fig 2a-c  Suppl. Fig. 3a-b  Suppl. Fig 4a-b  Suppl. Fig 5a-b  Suppl. Fig 6a-b | *pag.15*  *pag.16*  *pag.17*  *pag.18*  *pag.19* |

| **Section / Topic** | **No** | **CONSORT 2025 checklist item description** | **Reported on page no.** |
| --- | --- | --- | --- |
| **Title and abstract** | | |  |
| Title and structured abstract | 1a | Identification as a randomised trial | 1 |
|  | 1b | Structured summary of the trial design, methods, results, and conclusions | 2-3 |
| **Open science** | | |  |
| Trial registration | 2 | Name of trial registry, identifying number (with URL) and date of registration | 2 |
| Protocol and statistical analysis plan | 3 | Where the trial protocol and statistical analysis plan can be accessed | 23 |
| Data sharing | 4 | Where and how the individual de-identified participant data (including data dictionary), statistical code and any other materials can be accessed | 24 |
| Funding and conflicts of interest | 5a | Sources of funding and other support (e.g., supply of drugs), and role of funders in the design, conduct, analysis and reporting of the trial | 1 |
|  | 5b | Financial and other conflicts of interest of the manuscript authors | 1 |
| **Introduction** | | |  |
| Background and rationale | 6 | Scientific background and rationale | 4-5 |
| Objectives | 7 | Specific objectives related to benefits and harms | 5 |
| **Methods** | | |  |
| Patient and public involvement | 8 | Details of patient or public involvement in the design, conduct and reporting of the trial | 6 |
| Trial design | 9 | Description of trial design including type of trial (e.g., parallel group, crossover), allocation ratio, and framework (e.g., superiority, equivalence, non-inferiority, exploratory) | 6 |
| Changes to trial protocol | 10 | Important changes to the trial after it commenced including any outcomes or analyses that were not prespecified, with reason | 11-12 |
| Trial setting | 11 | Settings (e.g., community, hospital) and locations (e.g., countries, sites) where the trial was conducted | 6 |
| Eligibility criteria | 12a | Eligibility criteria for participants | 6 |
|  | 12b | If applicable, eligibility criteria for sites and for individuals delivering the interventions (e.g., surgeons, physiotherapists) | NA |
| Intervention and comparator | 13 | Intervention and comparator with sufficient details to allow replication. If relevant, where additional materials describing the intervention and comparator (e.g., intervention manual) can be accessed | 7, 8 |
| Outcomes | 14 | Pre-specified primary and secondary outcomes, including the specific measurement variable (e.g., systolic blood pressure), analysis metric (e.g., change from baseline, final value, time to event), method of aggregation (e.g., median, proportion), and time point for each outcome | 8-9 |
| Harms | 15 | How harms were defined and assessed (e.g., systematically, non-systematically) | 8 |
| Sample size | 16a | How sample size was determined, including all assumptions supporting the sample size calculation | 11-12 |
|  | 16b | Explanation of any interim analyses and stopping guidelines | NA |
| Randomisation: |  |  |  |
| Sequence generation | 17a | Who generated the random allocation sequence and the method used | 7 |
|  | 17b | Type of randomisation and details of any restriction (e.g., stratification, blocking and block size) | 7 |
| Allocation concealment mechanism | 18 | Mechanism used to implement the random allocation sequence (e.g., central computer/telephone; sequentially numbered, opaque, sealed containers), describing any steps to conceal the sequence until interventions were assigned | 7 |
| Implementation | 19 | Whether the personnel who enrolled and those who assigned participants to the interventions had access to the random allocation sequence | 7 |
| Blinding | 20a | Who was blinded after assignment to interventions (e.g., participants, care providers, outcome assessors, data analysts) | 7 |
|  | 20b | If blinded, how blinding was achieved and description of the similarity of interventions | NA |
| Statistical methods | 21a | Statistical methods used to compare groups for primary and secondary outcomes, including harms | 12 |
|  | 21b | Definition of who is included in each analysis (e.g., all randomised participants), and in which group | 12 |
|  | 21c | How missing data were handled in the analysis | 12 |
|  | 21d | Methods for any additional analyses (e.g., subgroup and sensitivity analyses), distinguishing prespecified from post-hoc | 10 |
| **Results** | | |  |
| Participant flow, including flow diagram | 22a | For each group, the numbers of participants who were randomly assigned, received intended intervention, and were analysed for the primary outcome | Fig. 1 |
|  | 22b | For each group, losses and exclusions after randomisation, together with reasons | 13-14, Fig. 1 |
| Recruitment | 23a | Dates defining the periods of recruitment and follow-up for outcomes of benefits and harms | 13 |
|  | 23b | If relevant, why the trial ended or was stopped | NA |
| Intervention and comparator delivery | 24a | Intervention and comparator as they were actually administered (e.g., where appropriate, who delivered the intervention/comparator, how participants adhered, whether they were delivered as intended [fidelity]) | 13-14 |
|  | 24b | Concomitant care received during the trial for each group | NA |
| Baseline data | 25 | A table showing baseline demographic and clinical characteristics for each group | Table 1 |
| Numbers analysed,  outcomes and estimation | 26 | For each primary and secondary outcome, by group:   - the number of participants included in the analysis - the number of participants with available data at the outcome time point - result for each group, and the estimated effect size and its precision (such as 95% confidence interval) - for binary outcomes, presentation of both absolute and relative effect size | 14, 15-16 |
| Harms | 27 | All harms or unintended events in each group | 14-15 |
| Ancillary analyses | 28 | Any other analyses performed, including subgroup and sensitivity analyses, distinguishing pre-specified from post-hoc | 16-17, 18 |
| **Discussion** | | |  |
| Interpretation | 29 | Interpretation consistent with results, balancing benefits and harms, and considering other relevant evidence | 19,20, 21 |
| Limitations | 30 | Trial limitations, addressing sources of potential bias, imprecision, generalisability, and, if relevant, multiplicity of analyses | 22, 23 |

**CONSORT 2025 checklist of information to include when reporting a randomised trial***

*We strongly recommend reading this statement in conjunction with the CONSORT 2025 Explanation and Elaboration and/or the CONSORT 2025 Expanded Checklist for important clarifications on all the items. We also recommend reading relevant CONSORT extensions. See [www.consort-spirit.org](http://www.consort-spirit.org).

Citation: Hopewell S, Chan AW, Collins GS, Hróbjartsson A, Moher D, Schulz KF, et al. CONSORT 2025 Statement: updated guideline for reporting randomised trials. BMJ. 2025; 388:e081123. <https://dx.doi.org/10.1136/bmj-2024-081123>.

© 2025 Hopewell et al. This is an Open Access article distributed under the terms of the Creative Commons Attribution License (<https://creativecommons.org/licenses/by/4.0/>), which permits unrestricted use, distribution, and reproduction in any medium, provided the original work is properly cited.

Study Protocol

**Primary HPT in individuals aged 65 years or older:**

**A prospective randomised study of surgical treatment versus observation**

*ClinicalTrials.gov Identifier: NCT01087619*

*Ethical approval: Regional Ethical Committee at Lund University; 2010/51 and 2017/211 (revision)*

**Background**

Primary HPT is a common condition among elderly individuals, particularly women. Swedish studies estimate the prevalence among postmenopausal women to be 3.1%. Most patients have mild disease with few or diffuse symptoms.

In the national quality register for thyroid and parathyroid surgery, the median age of patients is 62 years; 41% are 65 years or older, and 18% are 75 years or older. Of this group, 80% are women. The median level of ionised calcium among patients aged 65 or older is 1.46 mmol/L (albumin-corrected total calcium 2.78 mmol/L), and the 90th percentile for ionised calcium is 1.61 mmol/L (albumin-corrected total calcium 3.1 mmol/L). Thus, only about 10% of patients have high calcium levels according to conventional assessment.

It is relatively uncontroversial that patients under 50 years of age should be offered surgical therapy (1). Other proposed indications include a T-score below –2.5 SD (osteoporosis) on bone density, markedly increased urinary calcium excretion, clearly impaired renal function, and calcium values that are more than moderately elevated (1).

However, it is known that both symptomatology and organ complications correlate poorly with the actual calcium level.

Several epidemiological studies, particularly from Scandinavia, have shown an increased risk of cardiovascular morbidity and mortality (2), and this risk has been considered to decrease following surgery (3).

In a prospective randomised study of mild pHPT in patients aged <50–80 years, no significant difference in cardiovascular risk profile could be demonstrated after surgery, nor any increased mortality (4).

Patients with pHPT experience diffuse symptoms and reduced quality of life (5). However, in a recently published study comparing surgical treatment with observation alone, no difference could be shown between surgically treated patients and those who were only followed (control group) (6).

Our research group has shown that patients with pHPT experience improvement in bone density (7), which is an important factor in predicting future fracture risk. Furthermore, a recently published observational study found that fracture risk decreased in the group of patients undergoing surgical treatment compared with observation (8). A Danish cohort study (in-hospital patient register study) found that fracture risk in pHPT was increased up to 10 years before diagnosis, after which it normalised following surgery (9, 10).

Finally, a randomised study of patients aged 50–80 years with mild pHPT has demonstrated a small increase in bone density over a two-year period (6).

All these studies, however, are associated with potential bias, and the situation must still be regarded as uncertain, particularly in older age groups.

For elderly patients, the value of a T-score below –2.5 SD as an indication for pHPT surgery is uncertain, since the T-score is age-dependent. Thus, a large proportion of patients with pHPT would meet this criterion for surgery if one simply waited long enough; for example, 2–3% suffer from osteoporosis at age 50, compared with 50% at age 80. In addition, other factors influence future fracture risk, such as previous fractures, parent´s hip fracture, smoking, corticosteroid medication, rheumatoid arthritis, other secondary causes of osteoporosis, and alcohol intake (FRAX^®^ Fracture Assessment Tool). One study also shows that patients under 50 years of age have a higher risk of developing elevated calcium levels and/or organ complications compared with older patients (11).

We therefore intend to study surgical treatment versus clinical follow-up in a prospective randomised trial of sporadic primary HPT in patients aged 65 years and older, referred for evaluation for a first surgical intervention for the condition, and with ionised calcium <1.50 mmol/L. This subgroup currently represents approximately 50% of patients undergoing surgery for primary HPT.

The primary endpoint (on which the power calculation is based) will be the change in bone density over two years. Biochemical and clinical risk factors for cardiovascular disease will also be analysed, as well as renal function and cognitive function.

Patients randomised to the observation group will be followed with measurements of ionised calcium every three months. If the ionised calcium level during the study rises above 1.50 mmol/L (confirmed by repeated testing at least one week apart), the patient will be advised to undergo parathyroidectomy. Furthermore, patients who require specific medical treatment due to hypercalcemia, or who exhibit a Z-score of less than −2.5 SD during follow-up, will be recommended parathyroidectomy

Patients who, prior to randomisation, have 25(OH) vitamin D levels below 50 nmol/L will receive treatment for this.

The study lasts for two years. After two years, patients who have only been observed will be offered surgery if they so wish, or continued follow-up by an endocrinologist.

**Inclusion Criteria**

Patients with primary HPT undergoing first-time surgery for sporadic disease and aged 65 years or older.

**Exclusion Criteria**

• Z-score less than −2.5 SD regardless of site
• Hypercalcaemia requiring specific medical treatment
• Ionised calcium > 1.50 mmol/L
• Patients treated with lithium therapy
• Inability to understand information and/or to participate in planned follow-up

**Study Plan**

Patients referred to the Department of Surgery, Skåne University Hospital-Lund, for parathyroidectomy fulfilling inclusion criteria, and without exclusion criteria, will be offered participation in the study.

Before the outpatient visit, the following biochemical variables are analysed:
• P-Calcium
• Albumin
• S-Ionised calcium
• S-Creatinine
• P-PTH
• 24h urinary calcium excretion
• S-25 (OH) vitamin D_3_

Patients showing an S-25 (OH) vitamin D_3_ level below 50 nmol/L (a level at which PTH increases compensatory) will receive medical treatment with T. Tillval D® (10 µg D3, 400 IU), 1x2, with monitoring of calcium and PTH.

**Randomisation**

Patients receive written and verbal information about the study. After informed consent is obtained, randomisation is performed by drawing sealed envelopes stored in blocks of six.

**Non-randomised Patients**

1. Patients not randomised due to patient preference or any other reason will undergo standard assessment and follow-up. The same study protocol as for randomised patients will be completed when applicable.
2. Patients meeting exclusion criteria will be registered with baseline biochemical and densitometry values.

**Pre- and Postoperative Study Variables**

(to be assessed at 1 and 2 years)

I. Study protocol for medical history (medication, diseases, fracture risk factors according to FRAX^®^)
II. Cognitive tests:
• MMSE (general cognitive assessment)
• AQT (cognitive speed)

• Carotid ultrasound with measurement of intima thickness and plaques
• Echocardiography
• 24-hour blood pressure monitoring
• Densitometry (DEXA)
• Oral glucose tolerance test
• Ankle–brachial index

**Biochemical analyses:**
• P-Calcium
• S-Ionised calcium
• P-PTH
• S-25 (OH) vitamin D
• S-1,25 (OH)₂ vitamin D
• 24h urinary calcium excretion
• 24 h urinary albumin excretion
• S-Creatinine
• S-Osteocalcin
• S-Crosslaps
• S-Urate
• P-Glucose
• P-C-peptide
• S-Insulin
• S-Triglycerides
• S-Cholesterol (total)
• Lipoprotein electrophoresis (HDL, VLDL)
• HbA1c
• APO A1
• APO B
• CRP
• Iohexol clearance
• NT-proBNP

**Follow-up of Non-operated Patients**

Non-operated patients are followed every three months with:
• S-Calcium
• S-Ionised calcium
• S-PTH

**Postoperative Follow-up**

Patients are followed up after 4–6 weeks with:
• P-Calcium
• S-Ionised calcium
• P-Albumin
• S-Phosphate
• P-PTH

**Outcomes**

1. The primary outcome is hip densitometry. The primary endpoint is the change in densitometry at the femoral neck over a two-year period.
2. Secondary outcomes are cognitive tests, biochemical variables that are established risk factors for cardiovascular disease, 24-hour blood pressure monitoring, echocardiography, and intima thickness.

**Addendum to the study protocol regarding secondary outcome measures (14 November 2017)**

Kidney function (GFR estimated using iohexol clearance and urinary albumin excretion).

**Sample Size**

Power calculations have been performed to detect a difference at alpha 0.05 and beta 0.80 between the treated and untreated groups.

According to calculations from the literature, one should be able to demonstrate a difference for the primary endpoint, bone density at the femoral neck, between treated and untreated patients with 80 patients in each arm (the surgically treated group increases in bone density by approximately 4% and a similar decrease in the untreated group).

The chosen sample size may also allow detection of differences (alpha 0.05, beta >0.80) between untreated and treated groups in S-Insulin, HDL, atherogenic index, and cardiac dysfunction as assessed by echocardiography.

**Amendments to study protocol regarding power calculations and primary endpoint of the study (November 14, 2017)**

With slow recruitment and with new information available regarding the precision of DEXA measurement of bone density at the hip, showing that total hip measurement may be preferred to femoral neck for longitudinal follow-up of bone density, a revision of the power calculation was performed after 40 included patients.

The revised power calculation was executed without any previous data analysis for the primary outcome by the research group and used an empirical one-sample standard deviation estimate from the 40 patients randomized patients, without considering the randomization outcomes.

1. The observed baseline standard deviation for the 40 patients evaluated by DEXA as total hip was 0.14 g/cm2, and the mean bone density (measured as total hip) was 0.8919 g/cm2. To show a five per cent increase after two years in bone density for the intervention group and a similar decrease in the control group, with 80 per cent power and on the five per cent significance level, 42+42 patients is sufficient to evaluate bone density at the hip as the primary outcome, with the change of total hip bone density as the primary endpoint.
2. The lumbar spine (L1-L4) is reported as a secondary outcome.

The revised power calculation and the rational for performing this, was approved by the Regional Ethical Committee at Lund University; 2017/211.

**Patient Safety**

Patients in the observation group are monitored with calcium and PTH every three months.

1. Ionised calcium >1.50 mmol/L (confirmed at least one week apart) during follow-up constitutes a basis for recommending parathyroidectomy.
2. Patients in the untreated group who develop a Z-score < –2.5 SD at the femoral neck during follow-up will be recommended parathyroidectomy.
3. Hypercalcaemia requiring specific medical treatment constitutes grounds for parathyroidectomy.

**Registration, Publication and Presentation**

The study will be registered online according to established practice.

Publication will take place in an international journal, both regarding the primary outcome and the secondary outcomes.

Results will also be presented at national and international meetings.

**References**

1. AACE/AAES Task force on Primary Hyperparathyroidism. AACE/AAES position statement on the diagnosis and management of primary hyperparathyroidism. Endocrin Pract 2005, 11: 49-54

2. Lundgren E et al. Increased cardiovascular mortality and normalized serum calcium in patients with mild hypercalcaemia followed for 25 years. Surgery 2001; 130: 978-985

3. Hedbäck et al. The influence of surgery on the risk for death in patients with primary hyperparathyroidism. World J Surg 1991; 15: 399-405

4. Bollerslev et al. Effect of surgery on cardiovascular risk factors in mild primary hyperparathyroidism. J Clin Endocrinol Metab 2009; 94: 2255-2261

5. Lundgren E et al. Case-control study on symptoms and signs of “asymptomatic” primary hyperparathyroidism. Surgery 1998; 124: 980-985

6. Bollerslev et al. Medical observation, compared with parathyroidectomy, for asymptomatic primary hyperparathyroidism: A prospective, randomized trial. J Clin Endocrinol Metab 2007; 92: 1687-1692

7. Nordenström et al. Recovery of bone mineral density in 126 patients after surgery for primary hyperparathyroidism. World J Surg 2004; 28: 502-507

8. Lindi H et al. Effect of bone mineral density and parathyroidectomy on fracture risk in primary hyperparathyroidism. World J Surg 2009; 33: 406-411

9. Vestergaard P et al. Cohort study of risk for fracture before and after surgery for primary hyperparathyroidism. British Medical Journal 2000; 321: 598-602

10. Vestergaard P et al. Parathyroid surgery is associated with a decreased risk for hip and upper arm fractures in primary hyperparathyroidism: a controlled study. J Intern Med. 2004; 255: 108-114

11. Silverberg S et al. Age as criterion for surgery in primary hyperparathyroidism. Am J Med. 2002; 113: 681-684

**Supplementary Figures and Tables**

Suppl. Table S1

Baseline clinical and biochemical variables and densitometry in 16 randomised patients that did not complete the study and the 86 patients that completed the two-year follow-up. Mean (s.d.) is shown if not stated otherwise.

| Variable | Patients that did not complete the study  N=16 | Patients that completed the study  N=86 |
| --- | --- | --- |
| Randomised group, surgery: observation | 9:7, | 43:43, |
| Sex, women: men | 13: 3 | 65:21 |
| Age, years | 73.3(5.9) | 71.9 (4.2) |
| BMI | 28.3 (3.3) | 28.8 (5.0) |
| Vitamin D supplements, yes: no | 2: 14 | 21:65 |
| Bisphosphonate, yes: no | 0: 16 | 8:78 |
| Oral steroid treatment, yes: no | 0: 16 | 1:85 |
| Diabetes mellitus, yes: no | 6: 10 | 13:73 |
| Previous fracture, yes: no | 6: 16 | 30:56 |
| Total hip g/cm^2^ | 0.848 (0.099), n=15 | 0.867 (0.137), n=83 |
| Total Hip T-score | -1.4 (0.8), n=15 | -1.3 (1.0), n=83 |
| Total Hip Z-score | -0.2 (0.8), n=15 | -0.1(0.9), n=83 |
| Lumbar spine, g/cm2 | 1.069 (0.208) | 1.096 (0.202) |
| Lumbar Spine T-score | -1.0 (1.6) | -0.8 (1.6) |
| Lumbar Spine Z-score | 0.1 (1.5) | 0.3 (1.4) |
|  |  |  |
| Ionised Calcium (mmol/L) | 1.40 (0 .06) | 1.40 (0.05) |
| Total Calcium (mmol/L) | 2.62 (0.11) | 2.61(0.09) |
| PTH (pmol/L) | 11.1 (3.2) | 9.9 (2.9) |
| Phosphate (mmol/L) | 0.88 (0.15) | 0.82 (0.14) |
| Creatinine (umol/L) | 80 (23) | 76 (19) |
| Osteocalcin (ug/L) | 42 (33), n=15 | 33 (17), n=85 |
| Cross laps (ng/L) | 538 (271) | 532 (248), n=84 |
| 25 OH Vitamin D_3_ (nmol/L) | 55 (17) | 58 (22) |
| 1.25 (OH)_2_ Vitamin D_3_ (pmol/L) | 98 (30), n=13 | 97 (42), n=81 |
| Urinary calcium mmol/L/24 h | 4.19 (2.72), n=15 | 4.48 (2.98) |
| Urinary calcium mmol/L >6,25 in women and men > 7,49 mmol/L, yes: no | 3:12, n=15 | 19:63, n=82 |

Supplement Table S2

Change in BMD in per cent at the end of the 2-year study period for men and women, in the intervention group and control group, respectively. Mean and standard deviation (s.d.) are shown.

**Men**

Variable	Observation	Total Hip %	P-value

mean (s.d.)

Intervention	9	1.4 (6.6)

Control	11	-2.3 (3.4)	0.163

__________________________________________________________________________________

Variable	Observation	Lumbar spine %	P-Value

mean (s.d.)

Intervention	9	3.9 (4.6)

Control	12	0.7 (4.2)	0.125

======================================================================

**Women**

Variable	Observation	Total Hip %	P-value

mean (s.d.)

Intervention	32	1.3 (4.3)

Control	29	-1.1 (3.9)	0.029

__________________________________________________________________________________

Variable	Lumbar spine %

mean (s.d.)

Intervention	34	4.7 (5.2)

Control	31	-0.0 (4.2)	0.001

Suppl. Figure S1a-c.

Bone mineral density (BMD) at the femoral neck as analysed by mixed model repeated measurements ANOVA for the two groups of patients: (a) BMD (g/cm^2^); (b) Z-scores; (c) T-scores. Mean and s.d. are shown and p-value at the end of the study period. FU= follow-up (years)

a.

b.

c.

Suppl. Figure S2a-c.

Bone mineral density (BMD) at the distal radius as analysed by mixed model repeated measurements ANOVA for the two groups of patients: (a) BMD (g/cm^2^); (b) Z-scores; (c) T-scores. Mean and s.d. are shown and p-value at the end of the study period. FU=follow-up (years)

a.

b.

c.

**Suppl. Fig. S3**

BMD (g/cm2) analysed by mixed-model repeated-measures ANOVA for the two groups of patients, including only patients investigated with the same GE-Lunar equipment. Mean and 95 % confidence interval (c.i.) are shown. P-values at the end of the study period. FU=follow-up (years); Red line=intervention; Blue line= control.

Total hip	Lumbar Spine

**Suppl. Fig. S4**

BMD Z-score analysed by mixed-model repeated-measures ANOVA for the two groups of patients, including only patients investigated with the same GE-Lunar equipment. Mean and 95 % confidence interval (c.i.) are shown. P-values at the end of the study period. FU=follow-up (years); Red line=intervention; Blue line= control

Total Hip	Lumbar Spine

**Suppl. Fig. S5**

BMD T-score analysed by mixed-model repeated-measures ANOVA for the two groups of patients, including only patients investigated with the same GE-Lunar equipment. Mean and 95 % confidence interval (c.i.) are shown. P-values at the end of the study period. FU=follow-up (years); Red line=intervention; Blue line= control

Total hip	Lumbar Spine

**Suppl. Fig. S6**

FRAX^®^ analysed risk for fractures within a 10-year period by mixed-model repeated-measures ANOVA for the two groups of patients, including only patients investigated with the same GE-Lunar equipment. FU=follow-up (years); Red line=intervention; Blue line= control

Risk for hip fracture	Risk for any major osteoporotic fracture
